# The impact of COVID-19 induced emergency remote instruction on students’ academic performance at an HBCU

**DOI:** 10.1371/journal.pone.0264947

**Published:** 2022-03-10

**Authors:** Sayed Mostafa, Kathy Cousins-Cooper, Barbara Tankersley, Shea Burns, Guoqing Tang

**Affiliations:** Department of Mathematics & Statistics, College of Science & Technology, North Carolina A&T State University, Greensboro, North Carolina, United States of America; Flinders University, AUSTRALIA

## Abstract

The outbreak of the COVID-19 pandemic early in 2020 forced universities to shut down their campuses and transition to emergency remote instruction (ERI). Students had to quickly adapt to this new mode of instruction while dealing with all other distractions caused by the pandemic. This study integrates extensive data from students’ institutional records at a large Historically Black College and University (HBCU) institution with data from a students’ survey about the impact of COVID-19 on learning during the Spring 2020 semester to examine the impact of the transition to ERI on students’ performance and identify the main factors explaining variations in students’ performance. The main findings of our analysis are: (a) students’ university experience was positively correlated with performance (continuing students who spent at least one academic year at the university prior to the outbreak had better performance than freshman and new transfer students), (b) students’ perceived change in performance after the transition was positively associated with actual performance (students who perceived a decline in their performance after transition to ERI had significantly worse performance than other students), and (c) students’ prior online learning experiences and students’ emotional experiences with the COVID-19 disease were not significantly associated with performance. These results suggest that the approaches adopted by higher education institutions to support students during times of crisis should pay special attention to certain groups of students.

## Introduction

The outbreak of COVID-19 (caused by SARS-CoV-2) pandemic early in 2020 has impacted all aspects of our life, including the way of learning and teaching. To control the spread of this deadly virus, health officials have called for social distancing and self-isolation which have led to a large-scale lockdown forcing schools, colleges, and universities to close their campuses and transition to Emergency Remote Instruction (ERI) in the middle of the Spring 2020 semester. Under ERI–a sudden, temporary shift of course instructional delivery to remote delivery in response to a crisis that prevents face-to-face instruction–students had to complete their courses remotely. Courses offered under ERI are quite different from online courses that are designed to be delivered remotely in advance (e.g., [1]). While a large body of literature on students’ learning and performance in a well-planned online learning environment existed prior to the COVID-19 pandemic, nothing was known about students’ learning or performance under ERI. This situation has motivated many researchers to examine the impact of ERI on students’ learning and performance, leading to many articles published on this topic in just a few months after the outbreak (see [2–4] and references therein). As noted by Aristovnik et al. [2], most of these studies focused on a single country, single higher education institution, and/or single academic discipline.

In the United States, the COVID-19 pandemic had impacted individuals from minority groups, particularly African Americans and Hispanics, disproportionately in many ways. On the health side, African Americans were at higher risk of contracting and dying from COVID-19 due to health disparities [5]. The statistics of the Centers for Disease Control and Prevention (CDC) show that during the first wave of the pandemic, the Non-Hispanic Black/African American and the Hispanic populations had experienced hospitalization and death rates that are way above their share of the U.S. population [6, 7]. Therefore, students at Historically Black Colleges and Universities (HBCUs) and Minority Serving Institutions (MSIs) are likely to have been affected by COVID-19 more severely than others due to them having to deal with elevated levels of psychological, mental, and financial stress. *To the best of our knowledge*, *none of the studies on student learning during the COVID-19 pandemic thoroughly examined the learning experiences of underrepresented minority students with ERI during the COVID-19 pandemic*.

In this study, we use data from the largest HBCU institution in the United States, to (i) examine the variations in students’ experiences and performance during the transition to ERI; and (ii) identify the factors that explain/correlate with these variations. Specifically, the study addresses the following research questions:

1. How did variations in the performance of continuing students compare to first-year students?
2. How did variations in the performance of students with prior online learning experience compare to those without such experience?
3. How did variations in the performance of students with friends and/or family members infected by or dying from COVID-19 compare to those students without such experiences?

By examining the variations in students’ experiences and academic performance during the transition to ERI and identifying the factors that explain these variations at a large minority serving institution of higher education, this study makes critical contribution towards reaching more comprehensive understanding of the impacts of the pandemic on college students. Given that the minority groups of the U.S. population are likely to experience more harsh impacts from a pandemic as witnessed in the case of COVID-19, it is important for the higher education sector to understand how these disproportionate impacts are reflected on minority students. The findings of this study can be useful in guiding the formulation of interventions that help minority serving institutions better support their students during times of crisis such as pandemics. The study contributes to theory development through building several models for exploring many potential correlates of students’ performance during ERI such as students’ personal experiences with the COVID-19 disease, perceptions of own performance and instructors support during the transition to ERI, changes in study environment and financial situation due to the pandemic and prior experience with online learning, while controlling for students’ demographics and precollege preparation.

## Related literature

Shifting to online instruction and study from home due to the lockdown mandated by the COVID-19 pandemic was associated with several challenges related to students’ mental health and assessment (e.g., [8]). Mishra et. al [9] pointed to the importance of the use of counseling services to support the mental health and well-being of students during the pandemic. Detwiler [10] reports that a student’s performance is less dependent on the course delivery mechanism than on student’s motivation, maturity, and time management skills. Thus, whether a student performs well in the online setting may depend on intrinsic factors as well as the student’s prior experiences. With this in mind, our analysis of the impact of ERI on students’ performance examines two types of factors: (i) factors that were acknowledged prior to the pandemic such as self-efficacy, students’ university experience (new students versus continuing students), and prior experience with online learning; and (ii) factors instigated or affected by the pandemic such as students’ emotional experiences with the COVID-19 disease, students’ perceptions of instructors support, and other extrinsic factors including changes in financial situation and study environment due to the pandemic.

### Self-efficacy theory

Bandura [11] developed the theory of self-efficacy as an extension from social cognitive theory to explain how individuals judge their own competencies or skills (see also [12]). Bandura described self‐efficacy as “the belief in one’s capabilities to organize and execute courses of action required to produce given attainments.” [11, p.3]. Self-efficacy has been related to persistence, tenacity, and achievement in educational settings [13–16]. There are several studies that have shown how self-efficacy is positively correlated with student motivation and performance. Yoshida et al. [17] found that students who were confident in their ability to solve difficult problems were more likely to be academically motivated because self‐efficacious students were motivated to persevere despite the academic challenges, while those with low self‐efficacy reported low motivation in completing difficult academic tasks. A study by Vancouver et al. [18] found a positive association between self‐efficacy and motivation in undergraduate students in higher education. Prat‐Sala and Redford [19] found that students who reported high academic self‐efficacy in reading and writing were more motivated to study in comparison to those with low self‐efficacy. Chang et al. [20] found that high self‐efficacy internet learners reported being more motivated to learn in comparison to those with low self‐efficacy.

Perceptions of self-efficacy are derived from four sources of information: (a) personal accomplishments, (b) verbal persuasion, (c) vicarious learning experiences, and (d) physiological and affective reactions [13]. There have been studies that reinforce the impact that of these factors on self-efficacy, and in particular the factor of past experiences. Past experiences usually determine students’ perception of personal ability to learn and perform well in a subject. In a study conducted on a group of Chinese junior high school students, Gao [21] reported that students with either high or low self-efficacy not only experienced different degrees of exposure to the sources of self-efficacy but also held diverse opinions about the effects of each source on their mathematics self-efficacy. The researchers confirmed that girls recalled receiving more social persuasion and experiencing more anxiety than boys in learning mathematics, but girls’ perceptions of the influence of each source were not significantly different from boys. In Kalender et al. [22], the authors surveyed approximately 1400 students in a calculus-based physics course to investigate students’ motivational beliefs in physics using a validated survey. The findings of the study revealed that the gender differences in conceptual post-test performance were mediated by the model variables and that mathematics skills and prior physics preparation appears to be correlated with the large differences in self-efficacy, and that prior mathematics and physics learning appears to play a small direct role in shaping later physics learning outcomes but plays an indirect role in shaping physics learning outcomes via undermining or supporting student self-efficacy, which then itself influences learning. Robinson et al. [23] described a study that examined roles of two competence beliefs, self-efficacy for scientific tasks and science academic self-efficacy, during the final year of college. The authors indicated that females reported lower academic self-efficacy, despite having similar levels of prior achievement and outcomes and that structural relations also appeared to vary by gender. These results extend theoretical understanding of the roles of two distinct forms of self-efficacy and the potential mechanisms explaining gender gaps in science fields.

The aforementioned studies show that self-efficacy is based on previous experiences, and the more positive the previous experiences are, the higher self-efficacy is. Here we examine the performances of students with previous experiences (such as continuing students and students with prior online learning experience) and compare their performance to those students with less experience (such as first-year/ transfer students and students with no prior online learning experience). Self-efficacy is the underpinning of the proxy variables of student status and prior online learning experience included in our analyses.

### Performance of new students versus continuing students

Based on changes discussed in the previous studies of self-efficacy levels among first-year students, we examine research on the performance of new students versus continuing students. In a study comparing transfer students from two-year colleges to first-year students directly from high school in a kinesiology course at the University of Geulph-Humber in Ontario, Acai & Newton [24] found that females are more likely to enter college directly from high school while more males are more likely to transfer from two-year colleges. They also found there was no significant difference between the students’ academic performance and learning approach. On the other hand, Stewart & Martinello [25] studied measures of academic success of transfer students from community colleges and from other universities to non-transfer students at Brock University in Ontario taking introductory undergraduate social science courses. Comparing course withdrawal rates, they reported there were no significant differences between the three types of students. They also reported community college transfer students’ final course grades were not statistically different from non-transfer students. However, university transfer students earned statistically higher final course grades than non-transfer students. Research studies on the performance of transfer students, in general, have produced mixed results. A study by Aulck and West [26] found similar grade performance in transfer students from community colleges and new first-year students while transfer students from four-year universities had higher grades. This study also found no compelling evidence of “transfer shock”, an initial decline in performance during the first year of transfer students. An earlier study by Cedja et al. [27] reported that this “transfer shock” can vary according to discipline. In fact, it was found that transfer students in non-STEM disciplines experienced an increase in grade performance and those in STEM disciplines experienced a decrease.

Using machine-learning to predict academic success of students at the American University of Nigeria, Yakubu and Abubakar [28] found that females were 1.2 times more likely to succeed academically than males and that juniors and seniors were more likely to achieve higher cumulative GPA. Kalman et al. [29] studied students’ perspectives of online learning in upper-level and lower-level chemistry courses at a four-year undergraduate university in Georgia during the Spring 2020 transition to online learning. They found that “there are many personal characteristics that were determined to be critical for a student to easily convert to online learning, including adaptability, dedication, and self-awareness” and that many of the upper-level students had the skills needed to adapt to the transition to online learning. In addition, they found that students believed having a daily routine, taking care of their mental health and regular check-ins with their classmates and professors are some techniques that contributed to their success online regardless of their level.

The noted fluctuations in first year performance from these groups of students during a non-pandemic year influenced the decision to not compare these groups to each other, but to question their performance from one semester to the next during the year that included the pandemic.

### Online experience and performance

It is well-documented that online learning can be challenging in general, and especially so for students with no prior experience learning online [30]. It is also known that many instructors perceive online instruction as inferior to traditional face-to-face instruction [30]. Thus, it is anticipated that students had varying experiences with the transition to ERI.

Research shows that students’ experience with online learning is mixed. In a study by Cybinski and Selvanathan [31] where university students studying statistics using a traditional learning environment and a more flexible learning environment where lectures were replaced with a computer-managed learning tool, students in the group studying in the technology-supported flexible learning environment experienced more assessment anxiety and consequently less enjoyment of the subject. In addition, McLaren [32] reports on a study which compared the performance and the persistence of students taught using traditional methods and students taught online. In this study, McLaren reveals that persistence between the two cases as measured by the final grade in the course for the students who persist is independent of the mode of instruction. In a study involving two hundred and fifty-six undergraduate and graduate students at a southeastern university, Wang et al. [33] reported that students with previous online learning experiences tended to have more effective learning strategies when taking online courses, and hence, had higher levels of motivation in their online courses. Chang et al. [20] reported on a study including 87 college students enrolled in an online course and concluded that students with high internet self-efficacy outperformed those with low internet self-efficacy on the final exam and were more confident in their ability to complete an online course. They also noted that there were significant gender differences in that males had a higher degree of internet self-efficacy and confidence than females. While DeTure [34] and Puzziferro [35] indicated that technology self-efficacy was a poor predictor of the final grade and satisfaction in online courses, other researchers have reported that technology self-efficacy is positively correlated with online learning performance [36, 37].

Research supports that there is a need for concern to our university population in terms of discipline, delivery, and disposition with respect to online learning. In a study of community college students, Wladis et al. [38] noted that an online environment more strongly affects the attrition rates in STEM courses than non-STEM courses and students enrolled in online STEM courses are more likely to withdraw than students enrolled in non-STEM courses. In addition, Hachey et al. [39] studied a group of community college students and found that online experience alone was not enough to predict the success of students in online courses and that students were successful in subsequent online STEM courses if they were successful in prior online courses regardless of their Grade Point Average (GPA). Cochran et al. [40] found that the cumulative GPA in prior online courses and class standing are significant to retention in online courses and that seniors were less likely to withdraw from online courses than non-seniors. Xu & Jaggars [41] found that all types of students experienced declines in performance in online courses but the students with the strongest declines were males, younger students, Black students, and those with lower GPA. Jaggars [42] argues that students prefer to take “easy” courses online and the “difficult” or “important” ones face-to-face as it can be harder to grasp the content of difficult courses from online learning where students are distracted by things that they may have at home like gaming consoles. Hachey et al. [39, 43] found that students with successful prior online experience have better success in subsequent online courses.

The pre-planned online learning setting considered in the above literature is fundamentally different from the emergency remote learning induced by the COVID-19 pandemic. Mishra et al. [9] studied students’ experiences with online learning during the pandemic and noted the following: internet access and connectivity issues were major challenges to online learning at the study institution; while some students found it difficult to pay attention during online classes, others liked having the ability to watch, pause, and re-watch videos; and some females reported having to deal with household responsibilities making it difficult to learn from home. Clark et al. [44] studied the academic performance of 1835 middle schoolers in the ninth grade at three middle schools in China on 11 exams. The data consisted of exam results prior to the COVID-19 pandemic and during lockdown with the three schools responding to student learning differently. They found that performance improved by about 23 points (a 0.20 standard deviation) for students who received online learning and additional support that included lesson videos created by the students’ own teachers. In addition, they found that students who received additional support and online lessons from high-quality teachers other than the students’ own teachers performed better than those who received lessons from their own teachers.

Motivated by this research, the current study aims to further investigate the association between students’ prior online learning experience and their performance during the pandemic.

### Emotional experiences with COVID-19 and performance

Statistics show that the COVID-19 pandemic had affected individuals from minority groups, particularly African Americans and Hispanics, disproportionately in many ways. On the health side, African Americans were at higher risk of contracting and dying from COVID-19 due to health disparities [5]. The statistics of the Centers for Disease Control and Prevention (CDC) show that during the first wave of the pandemic, the Non-Hispanic Black/African American and the Hispanic populations had experienced hospitalization and death rates that are way above their share of the U.S. population [6, 7]. These rates were even more troubling among individuals in the college age (15–24 years old) with the observed death rates being 27.75% for non-Hispanic white, 33.96% for Non-Hispanic Black/African American, and 35.85% for Hispanic/Latino [6, 7]. In a survey conducted among students at the study institution in the Spring 2020 semester, 21.82% of the student participants reported that they had family members infected by COVID-19; 15.24% reported that they had close friends infected by COVID-19, 9.65% reported that they had lost family members due to COVID-19, and 3.54% reported that they had lost close friends due to COVID-19. Did such experiences impact students’ academic performance? And if so, how? In this article, we try to address these questions via integrating students survey data with data from students’ academic records. By addressing these questions, the current study will complement the extant literature suggesting that, in general, students’ GPA tends to be negatively affected during the semester of their loss [45].

### Extrinsic factors

There are several other factors that may have impacted students’ academic performance after the transition to virtual instruction due to the outbreak. Although the focus of the study is put on the three factors described above as they map directly to our three research questions, the study accounts for the possible impacts of several extrinsic factors.

#### Financial situation

Economically, the financial aid statistics at HBCUs show that most minority students come from the low-income class of the U.S. population. For instance, more than 83% of the students at the study institution received financial aid during the Spring 2020 semester. The pandemic had likely worsened the financial situation of most minority students and their families. According to the survey conducted among students at the study institution in Spring 2020, 47.52% of students reported that their financial situation had worsened after the outbreak, 12% of students reported that they needed to purchase technology tools to take part in their classes after transitioning to virtual instruction, but they could not obtain them, and 11.51% of students reported that they had no proper internet access for virtual classes. It is well-documented in the literature that students with difficult financial situations are more likely to have lower academic performance [46, 47]. Specifically, females, first-generation, and minorities with financial stress, such as paying for college, buying books, and feeling their academics, have lower academic performances [47] (see also [48]). Thus, our analysis accounts for this factor via including students self-reported change in their financial situation after the outbreak and availability of technology tools necessary for virtual learning as well as their financial aid status obtained from students’ records.

#### Study environment

Attending classes and studying on campus is different from attending classes virtually and studying from home. While on campus, students generally receive academic motivation and help from their interactions with their peers and teachers. Before the pandemic, the students participants of the study used learning management systems such as Blackboard, MyLab Math, and WebAssign primarily to access syllabi and other course materials and complete homework assignments. The outbreak of COVID-19 and transition to virtual instruction forced students to learn and study in a totally new environment. Some courses were administered synchronously through technologically-mediated platforms such as Zoom and Blackboard Collaborate Ultra. Others were administered asynchronously through lectures prepared and posted on YouTube or Blackboard. In their study about online teaching/learning experiences during the COVID-19 pandemic, Mishra et al. [9] found that those faculty skilled in using social media apps such as Facebook, Twitter, and Instagram had relatively smoother transitions to using the online educational platforms for classes. Elci & Abubakar [49] pointed to the importance of the task-technology fit in supporting users to perform coursework during the COVID-19 pandemic. For many students, learning and studying from home can be much harder than learning and studying on campus. The geographical location of the home and the student’s responsibilities in the home can affect student’s performance and motivation. Mishra et al. [9] noted that students in urban areas performed better than those in rural areas who experienced unstable internet connections due to infrastructure. Also, students assigned household chores during lockdown experienced lack of motivation for their studies. Black students who stay on campus earn higher GPAs than students who live at home with their parents [50]. In a survey conducted among students at the study institution in Spring 2020, about 74% of students reported that their study environment had worsened after the transition to virtual instruction. In the analysis, we use students’ self-reported data to account for changes in study environment and safety of students’ living place after the outbreak.

### Demographic characteristics and other covariates

In addition to the factors described above, the analyses reported in this study account for students’ demographics such as gender, rurality of origin, residency status, and whether they used to live on-campus before the outbreak. Other factors included in the analyses are students’ satisfaction with instructors’ support provided during the transition to virtual instruction, as well as students’ college preparation metrics, namely, ACT/SAT scores and high school GPA.

## Methodology

### Data and participants

The study took place at a large public land grant HBCU and it was reviewed and exempted by the North Carolina A&T State University’s Institutional Review Board (IRB-19-0202). The data used in this study was obtained from student records and a student survey conducted among undergraduate students in Spring 2020. During the last two weeks of the Spring 2020 semester, students in 11 mathematics/statistics courses (41 sections) completed a Qualtrics survey (COVID Impact Survey) about the impact of COVID-19 on their learning during the Spring 2020 semester. A list of students’ email addresses was obtained from the sections instructors who also encouraged students in their sections to complete the survey by offering extra participation points as an incentive. The 41 sections included in the study were a convenience sample as those were the sections whose instructors agreed to share their students contact information to be invited for the survey. However, these 41 sections covered wide-range of lower- and mid-level mathematics and statistics courses that are taken by students from most majors on campus. Out of the 1,516 students who were invited via Qualtrics to take the survey, 792 completed the survey resulting a response rate of 52.24%. All survey respondents agreed to a consent statement prior to starting the survey. The questionnaire consisted of 35 questions including general demographic questions, questions about the financial and academic impact of COVID-19 on those students, and the students’ experiences during the transition to remote instruction after the outbreak. The data from the survey was supplemented with data obtained from students’ records (Office of Strategic Planning and Institutional effectiveness–OSPIE) which included baseline measures of pre-college preparation (such as high school GPA and standardized ACT/AST test scores), course enrollment data (such as count and level of courses the student was enrolled in during Fall 2019 and Spring 2020), and students’ academic achievement as measured by term GPA. The survey data was linked to the students’ records data and the final data was deidentified before conducting any analysis. To measure the impact of ERI on students’ performance, we used the change in students’ GPA between Fall 2019 (prior to COVID-19) and Spring 2020 (COVID-19 outbreak and transition to remote instruction). This led to a final sample size of *n =* 764 undergraduates who completed the COVID Impact Survey and were enrolled at the institution in both Fall 2019 and Spring 2020.

Table 1 provides the description, source, and summary statistics for the variables used in the analyses.

**Table 1:** Descriptions and summary statistics for the variables used in the analysis.

Variable	Description	Source	Mean (SD)/%	Range
***Dependent variable***				
GPA Change	Difference between SP20 Term GPA and FA19 Term GPA	OSPIE	-0.032 (0.674)	-2.783–2.590
***Baseline measures***				
ACT/SAT	Standardized ACT/SAT composite score. Most of students had SAT scores, but some students had ACT scores. Both ACT and SAT scores were changed to z-scores. Then, SAT z-scores were used for students with SAT scores available and ACT z-scores were used for those w/o SAT score.	OSPIE	0.047 (1.002)	-3.160–3.206
HS GPA	Weighted high school GPA.	OSPIE	3.724 (0.550)	1.158–6.120
***Demographic variables***				
Gender	Dummy variable coded as: Female = 0, Male = 1.	OSPIE	1 = 42.28%	0–1
Rural	Dummy variable coded as: No = 0 if student is not from a rural area, and Yes = 1 if otherwise.	OSPIE	1 = 20.42%	0–1
Residency	Dummy variable coded as: In-State = 0, Out-of-State = 1.	OSPIE	1 = 37.30%	0–1
Living On-campus	Were you living on-campus in Spring 2020 before the COVID-19 outbreak? A dummy variable coded as: No = 0, Yes = 1.	Survey	1 = 68.59%	0–1
Living Place Safety	Do you feel safe in current living place? A Likert scale variable coded as: Never = 1, Rarely = 2, Sometimes = 3, Often = 4, Always = 5.	Survey	4.69 (0.632)	1–5
Financial Aid SP20	Did you receive financial aid during Spring 2020? A dummy variable coded as: No = 0, Yes = 1.	Survey	1 = 83.09%	0–1
***Target explanatory variables***				
FA19 Enrollment Status	Categorical variable coded as: Continuing = 1 (reference category), New Freshman = 2, and New Transfer = 3. Two other categories with very few cases (0.52% Returning and 1.31% Unclassified) were omitted.	OSPIE	1 = 46.47%	1–3
			2 = 44.76%	
			3 = 6.94%	
Online Experience	Dummy variable coded as No = 0 if student had not taken any online course over the past 5 years, and as Yes = 1 otherwise.	OSPIE	1 = 26.70%	0–1
Infected By COVID	Were any of your family members or close friends infected by COVID-19? A categorical variable coded as: None = 1 (reference category), Close Friend(s) = 2; Family Member(s) = 3; Both Close Friends and Family Members = 4; Prefer Not to Answer = 5.	Survey	1 = 60.21%	1–5
			2 = 9.29%	
			3 = 16.89%	
			4 = 7.33%	
			5 = 6.28%	
Died By COVID	Did you lose any of your family members or close friends due to COVID-19? A categorical variable coded as: None = 1 (reference category), Close Friend(s) = 2; Family Member(s) = 3; Both Close Friends and Family Members = 4; Prefer Not to Answer = 5.	Survey	1 = 84.03%	1–5
			2 = 2.09%	
			3 = 8.38%	
			4 = 0.65%	
			5 = 4.84%	
***Control covariates***				
Hours Attempted Difference	Difference between number of hours attempted in SP20 and FA19.	OSPIE	0.203 (2.213)	-11–9
Internet Access	In your current place of residence, do you have proper internet access that you use for your online classes? A dummy variable coded as No = 0 and Yes = 1.	Survey	1 = 88.61%	0–1
Tech Availability	Did you need to purchase major technology tools (e.g., laptop) for online classes after the COVID-19 outbreak? A categorical variable coded as: No = 1, Yes, purchased tech tools = 2, Yes, but couldn’t acquire some = 3.	Survey	1 = 73.69%	1–3
			2 = 14.27%	
			3 = 11.91%	
***Other covariates***				
Instructors Support	Please rate the extent to which you agree with the following statement: “my instructors provided adequate support and understanding during the COVID-19 outbreak.” A Likert scale variable coded as: Strongly disagree = 1, Agree = 2, Neither agree nor disagree = 3, Agree = 4, Strongly agree = 5.	Survey	3.050 (1.084)	1–5
Financial Situation	Compared to the financial situation you had before the COVID-19 outbreak, how did your situation change? A categorical variable coded as: Didn’t change = 1 (reference category), Improved = 2, Worsened = 3.	Survey	1 = 46.60%	1–3
			2 = 6.28%	
			3 = 47.12%	
Study Environment	Compared to the study environment you had before the COVID-19 outbreak, how did your environment change? A categorical variable coded as: Didn’t change = 1 (reference category), Improved = 2, Worsened = 3.	Survey	1 = 19.76%	1–3
			2 = 6.02%	
			3 = 73.69%	
Performance Perception	How did your performance in the courses you are taking this semester [SP20] change after going online due to the COVID-19 outbreak? A categorical variable coded as: Didn’t change = 1 (reference category), Improved Overall = 2, Worsened Overall = 3.	Survey	1 = 23.43%	1–3
			2 = 15.05%	
			3 = 60.86%	
COVID News	About how much time do you spend daily reading/ watching/ listening to news about COVID-19? An ordinal variable coded as: <0.5 hour = 1; 0.5–1 hour = 2; 1–2 hours = 3; 2–3 hours = 4; >3 hours = 5.	Survey	1 = 42.41%	1–5
			2 = 35.86%	
			3 = 12.44%	
			4 = 4.45%	
			5 = 3.93%	

### Statistical analysis

To address the research questions of this study, we used a combination of graphical techniques, multivariable regression models and analysis of variance (ANOVA) models. To explain variations in the outcome variable (GPA Change), several regression models were fitted to the data from 666 students who completed the COVID Impact Survey in Spring 2020, were enrolled at the institution in both Fall 2019 and Spring 2020, had complete baseline data from students institutional records, and their Fall 2019 enrollment status was either continuing, new freshman or new transfer (students meeting these conditions are referred to as the “full sample” henceforth). The full sample models included (i) a full regression model for GPA change with all other variables in Table 1 included as explanatory variables or control covariates; and (ii) a reduced regression model for GPA change on a smaller set of variables selected from the full model variables by the stepwise variable selection algorithm as implemented by the stepAIC function in the MASS package of R [51]. This algorithm sequentially adds variables to the model and uses the change in the model’s Akaike Information Criterion (AIC) index to decide whether the added variable should be retained in the model. ANOVA models followed by post-hoc multiple comparisons were used to study differences in GPA change among different groups of students defined by select explanatory variables.

To investigate the sensitivity of results obtained from the full sample data (which contained students from a wide range of mathematics and statistics courses) to the study population, we repeated the full sample analysis on a subset of the full sample focusing only on students that were enrolled in a pre-calculus or a calculus course in Spring 2020 (*n* = 406). We also assessed the sensitivity of results obtained from the above complete-case analyses to survey nonresponse (recall that the COVID Impact Survey response rate was only 52.24%) by repeating the analysis on the data where survey responses were imputed for students who were invited to the survey but did not participate. We note that the data for the response variable (GPA change) and many of the covariates and explanatory variables were available for all students invited to the survey from students’ institutional records. Only the covariates/explanatory variables obtained from the survey had missing data for students who did not participate. The multiple imputation technique was used to impute the missing values for the survey variables and the regression analysis was performed on the resulting multiple “completed” datasets. We used the multivariate imputation by chained equations (MICE) approach of van Buuren [52] to impute missing values for the survey nonrespondents. This approach allows for imputing variables of different types (i.e., numerical and categorical). Imputation was conducted using the mice package in R [53] with predictive mean matching imputation for numerical variables, logistic regression imputation for binary variables, and polytomous regression imputation for nonbinary categorical variables. To ensure that the imputation model preserves the relationships of interest in the analysis model, as recommended in the multiple imputation literature (e.g., [54–56]), all remaining variables from the analysis model were included in the imputation model as predictors for the variable being imputed.

All analyses were conducted using the open-source statistical software R [57]. Results were considered statistically significant if the p-value was below the 0.05 significance level.

## Results

**Fig 1:**
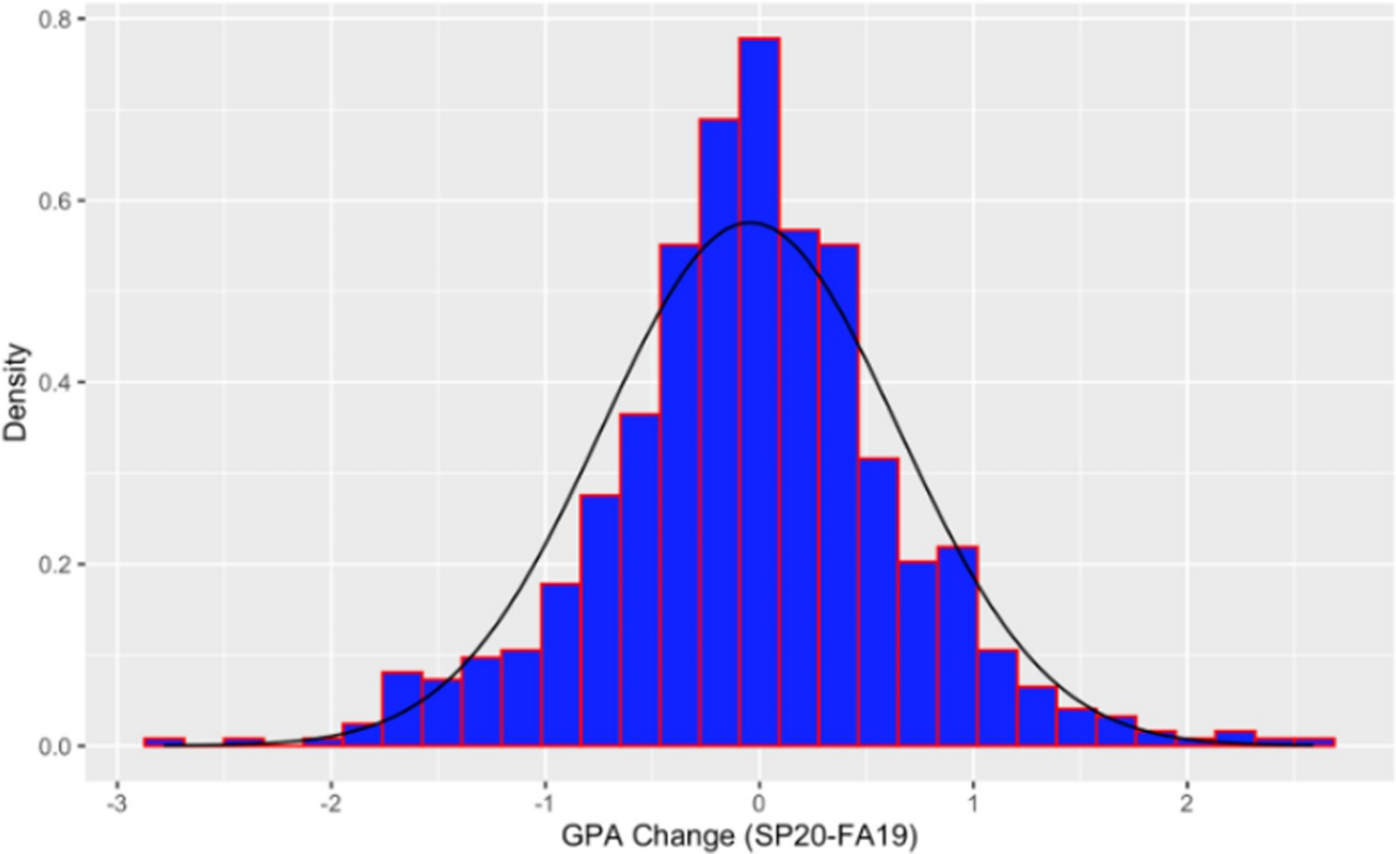
Full sample distribution of GPA change (outcome variable).

In this section, we report and discuss the results of our analyses. The analyses were focused on identifying the factors that may have affected STEM students’ performance during the transition to remote instruction after the COVID-19 outbreak. More specifically, the main goal of the analysis was to explain the changes in students’ GPA (SP20 –FA19) for different groups of students. The distribution of the outcome variable (change in GPA), with the normal probability distribution curve overlayed, is depicted in Fig 1. To verify validity of the regression model assumptions, residual analysis and model diagnostics were performed for each model we fitted, and all assumptions were found to be reasonably satisfied.

**Fig 2:**
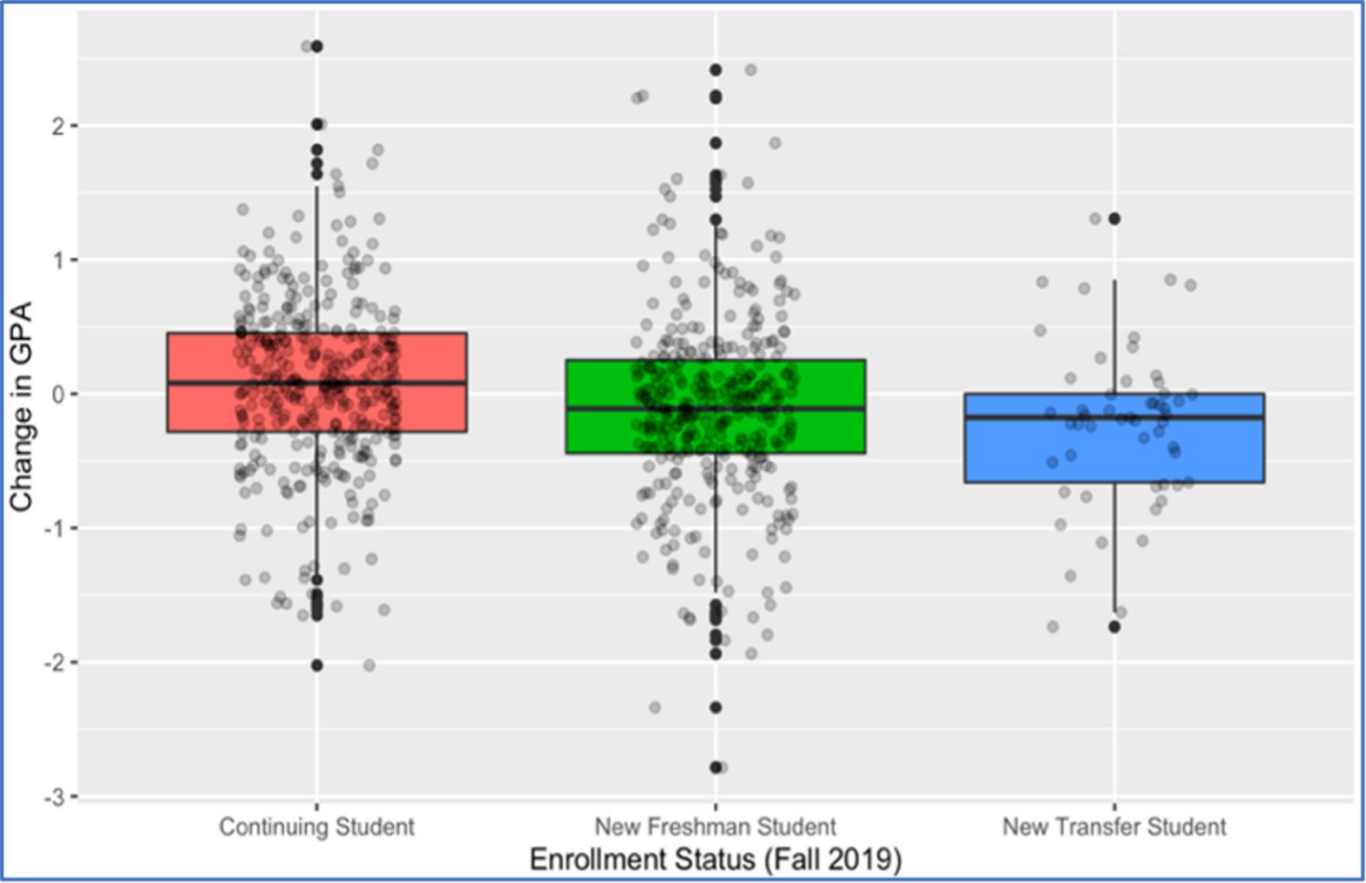
Distribution of GPA change by students’ enrollment status in FA 2019 [full sample].

Results of four regression models that we fitted for the outcome variable are reported in Table 2. The first column in Table 2 lists all explanatory variables and covariates included in the regression analysis. Considering the full sample of 666 students, Model I used all explanatory variables and covariates that may explain the outcome variable. In this model, the three variables ACT/SAT standardized score, FA19 enrollment status, and students’ perception about their performance during the transition to remote instruction were found to be significantly associated with the outcome variable as indicated by the corresponding low p-values. On the other hand, both previous online course experience and COVID-19 experiences (having close friends/family members infected by or dying from COVID-19) were not significantly associated with the change in GPA (p-values are well-above 0.05). Using the results of Model I, we can conclude that after accounting for all other predictors and covariates included in that model, on average, compared to continuing students (i) new freshmen students had about 0.228 lower improvement (or higher drop) in their GPA between Spring 2020 and Fall 2019; and (ii) transfer students had about 0.373 lower improvement (or higher drop) in their GPA between Spring 2020 and Fall 2019. This result is further illustrated in Fig 2. The third column in Table 2 displays the coefficients (p-values) of the variables selected by the stepwise variable selection algorithm applied to Model I. The resulting reduced model, labelled Model II in Table 2, shows that, in addition to the three significant predictors found in Model I, three other explanatory variables “Living Place Safety”, “Financial Aid SP20” and “Internet Access” may be important predictors as determined by the AIC, although not statistically significant at the 0.05 threshold. The ANOVA results reported in Table 3 –obtained from modeling the outcome variable on enrollment status in FA19 solely–confirm the importance of the students’ university experience as the main predictor of their change in GPA. The post-hoc pairwise comparisons, with Tukey adjustment to control the experiment-wise type I error rate at 0.05, confirm that both new freshmen and new transfer students had worse GPA change outcome than continuing students: difference in mean GPA change of -0.185 (p-value = 0.001) and -0.308 (p-value = 0.005), respectively. No statistically significant difference is found in GPA change between new freshmen and new transfer students (difference in mean GPA change is -0.122 with p-value = 0.428).

**Table 2:** Results of regression modeling [coefficient (p-value)] for change in GPA (before vs. during COVID-19).

Explanatory Variable	Full Sample (*n =* 666)		Calculus Sample (*n =* 406)	
	Model I	Model II	Model I	Model II
ACT/SAT	0.096 (**0.0038**)	-0.091 (**0.0010**)	0.076 (0.0941)	0.063 (0.1137)
HS GPA	-0.034 (0.5758)		-0.060 (0.4703)	
Gender: Male	-0.047 (0.4127)		-0.003 (0.9650)	
Rural: Yes	-0.057 (0.4383)		-0.069 (0.4953)	-0.133 (0.1411)
Residency: Out-Of-State	0.021 (0.7315)		0.041 (0.6146)	
Living On-campus: Yes	0.034 (0.5951)		0.073 (0.4316)	
Living Place Safety	0.068 (0.1504)	0.063 (0.1544)	0.089 (0.1598)	
Financial Aid SP20: Yes	0.110 (0.1467)	0.105 (0.1473)	0.157 (0.1391)	0.191 (0.0531)
FA19 Enroll. Status:				
Continuing (Ref.)				
New Freshman	-0.228 (**0.0004**)	-0.226 (**0.0001**)	-0.287 (**0.0026**)	-0.271 (**0.0008**)
New Transfer	-0.373 (**0.0041**)	-0.345 (**0.0059**)	-0.552 (**0.0071**)	-0.489 (**0.0122**)
Online Experience: Yes	-0.003 (0.9710)		0.013 (0.9010)	
Instructors Support	0.023 (0.3913)		-0.004 (0.9059)	
Study Environment:				
Didn’t change (Ref.)				
Improved	-0.023 (0.8572)		-0.002 (0.9889)	
Worsened	0.027 (0.7166)		-0.029 (0.7796)	
Financial Situation:				
Didn’t change (Ref.)				
Improved	-0.139 (0.2492)		-0.249 (0.1164)	
Worsened	0.002 (0.9679)		0.014 (0.8610)	
Performance Percept.:				
Didn’t change (Ref.)				
Improved	0.001 (0.9946)	-0.023 (0.7881)	-0.056 (0.6554)	-0.064 (0.5820)
Worsened	-0.213 (**0.0029**)	-0.202 (**0.0018**)	-0.236 (**0.0159**)	-0.267 (**0.0020**)
Hours Attempted Diff.	-0.003 (0.8041)		-0.009 (0.5877)	
Internet Access: Yes	-0.108 (0.2325)	-0.138 (0.1090)	-0.183 (0.1413)	-0.169 (0.1478)
Tech Availability:				
Had all tech (Ref.)				
Purchased Tech	0.033 (0.6950)		-0.048 (0.6947)	
Couldn’t Acquire Tech	0.110 (0.2328)		0.114 (0.3737)	
Infected By COVID:				
None (Ref.)				
Close Friend(s)	-0.048 (0.6198)		-0.041 (0.7831)	
Family Member(s)	0.031 (0.7184)		0.020 (0.8579)	
Both	-0.009 (0.9390)		-0.155 (0.3717)	
Prefer Not to Answer	0.031 (0.8252)		0.057 (0.7723)	
Died By COVID:				
None (Ref.)				
Close Friend(s)	-0.108 (0.5832)		-0.326 (0.2599)	
Family Member(s)	0.051 (0.6541)		-0.046 (0.7686)	
Both	0.359 (0.2844)		0.659 (0.1103)	
Prefer Not to Answer	-0.063 (0.6874)		-0.035 (0.8710)	
COVID News:				
<0.5 hour (ref.)				
0.5–1 hour	-0.084 (0.2519)		-0.044 (0.6589)	
1–2 hours	0.027 (0.7264)		0.114 (0.2836)	
2–3 hours	0.017 (0.8648)		0.006 (0.9654)	
>3 hours	0.058 (0.5223)		0.124 (0.3322)	
Constant	-0.068 (0.8496)	-0.053 (0.8173)	0.114 (0.8203)	0.334 (**0.0420**)
Adjusted R^2^ (F P-value)	0.03 (**0.0093**)	0.05 (<**0.0001**)	0.03 (0.0618)	0.05 (**0.0002**)

**Table 3:** ANOVA results for GPA change (before vs. during COVID-19) by enrollment status in FA 2019 [full sample].

FA 2019 Enrollment Status	Difference in Means	Tukey Adj. Confidence Interval (2.5%; 97.5%)	Tukey Adj. P-value
New Freshman–Continuing	-0.185	(-0.304; -0.067)	**0.001**
New Transfer–Continuing	-0.308	(-0.538; -0.077)	**0.005**
New Transfer–New Freshman	-0.122	(-0.353; 0.109)	0.428

ANOVA F-Statistic = 9.34 with p-value < 0.0001.

### Sensitivity of results to study population

**Fig 3:**
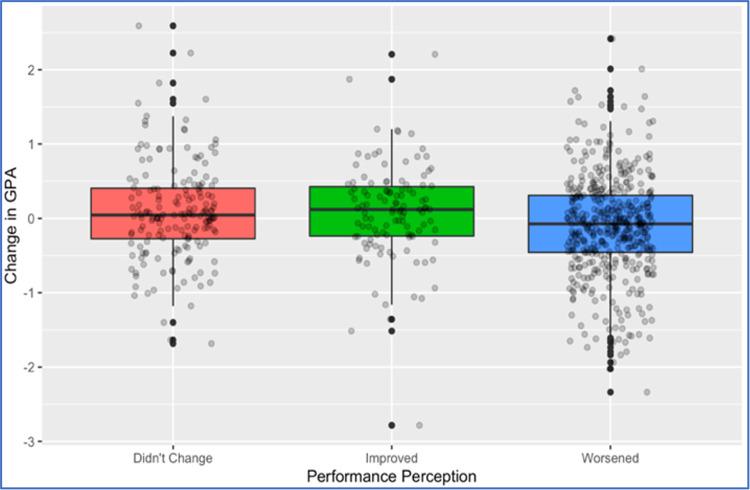
Distribution of GPA change by students’ perception about their performance after the outbreak [full sample].

**Fig 4:**
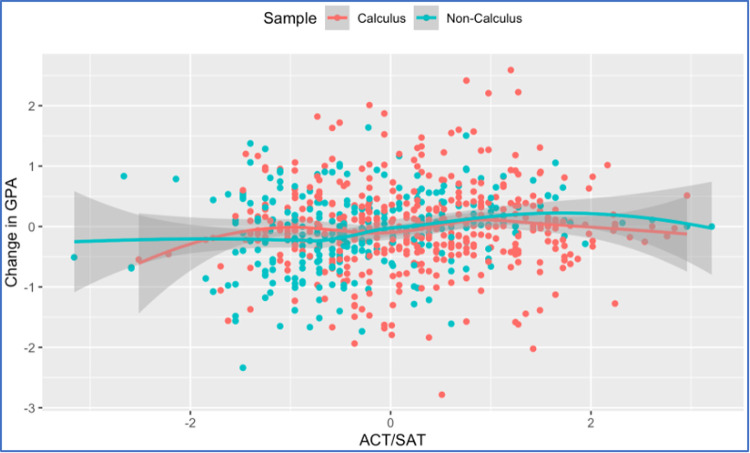
Relationship between GPA change and ACT/SAT scores by sample (calculus vs non-calculus).

The calculus sample results (from students enrolled in a pre-calculus or a calculus course in Spring 2020) are reported in the last two columns of Table 2 where we note that the students’ university experience (FA19 enrollment status) continues to be a significant predictor for change in students’ performance between Spring 2020 and Fall 2019. Table 4, showing the results of an ANOVA model between enrollment status and GPA change of students in the calculus sample, confirms this result. The pairwise comparisons reported in the same table indicate that new freshmen students had significantly worse GPA change than continuing students (difference in means of -0.200 with p-value = 0.013), whereas new transfer are not statistically significantly different from continuing students (p-value = 0.070) despite the larger mean difference observed in these two groups (-0.341). The insignificance of the latter difference might be attributed to the smaller size of the new transfer sample as compared to the new freshmen sample and/or different levels of variability in GPA change in the two samples. Students’ perception about performance during the transition was significant in both analyses (full sample and calculus sample) with students who perceived their course performance to have worsened after the outbreak experiencing significantly lower improvements or higher drops in their GPA between Spring 2020 and Fall 2019 as compared to other students. This result is further illustrated in Fig 3. On the other hand, students’ pre-college preparation (ACT/SAT) was not significant in any of the calculus sample models although being significant (but with very marginal effect as shown by the small coefficients) in the full sample models (see Fig 4 for a depiction of the relationship in both samples). For the calculus sample, we note that the full model (Model I) is not statistically significant (F P-value = 0.0618) despite the significance of the students’ enrollment status and performance perception terms, whereas the reduced model (Model II) is statistically significant (F P-value = 0.0002).

**Table 4:** ANOVA results for GPA change (before vs. during COVID-19) by enrollment status in FA 2019 [calculus sample].

FA 2019 Enrollment Status	Difference in Means	Tukey Adj. Confidence Interval (2.5%; 97.5%)	Tukey Adj. P-value
New Freshman–Continuing	-0.200	(-0.364; -0.035)	**0.013**
New Transfer–Continuing	-0.341	(-0.702; 0.021)	0.070
New Transfer–New Freshman	-0.141	(-0.497; 0.215)	0.621

ANOVA F-Statistic = 5.24 with p-value = **0.0056.**

In both sets of analyses, full sample and calculus sample, the comparisons stated in research questions 2 & 3 were not statistically significant. That is, our models show that, in terms of performance change (GPA change), students with previous online course experience were not significantly different from those without such experience, and that students who had close friends or family members contracting COVID-19 were not significantly different from those without such experiences.

### Sensitivity of results to survey non-response

Out of 1516 students who were invited to complete the COVID Impact Survey in Spring 2020, 1250 students were enrolled at the institution in both Fall 2019 and Spring 2020, had complete baseline data available from institutional records, and their Fall 2019 enrollment status was either continuing, new freshman or new transfer. Of those 1250, only 666 (53.28%) students completed the survey. Table 5 compares the characteristics of these 666 survey respondents to the nonrespondents using data available for all students from institutional records. These statistics show that survey respondents differed from the nonrespondents in the following ways: they had significantly lower drops in their GPA on average (respondents = -0.043 vs. nonrespondents = −0.344; p-value<0.0001), significantly higher average high-school GPA (respondents = 3.750 vs. nonrespondents = 3.570; p-value<0.0001), as well as significantly lower percentages of males (43.09% vs 54.79%) and in-state students (61.81% vs 71.40%). Compared to nonrespondents, respondents on average had attempted higher number of course hours in Spring 2020 versus Fall 2019. These results suggest that our models fitted to the respondents only data may benefit from imputing the survey items for nonrespondents and running a completed data analysis.

**Table 5:** Summary statistics of survey respondents and nonrespondents on data available from institutional records.

Variable	Respondents (*n =* 666)	Nonrespondents (*n =* 584)	P-value*
GPA Change (SP20 –FA19), Mean (SD)	-0.043 (0.693)	-0.344 (0.908)	**<0.0001**
ACT/SAT, Mean (SD)	0.067 (0.994)	0.051 (0.948)	0.8000
HS GPA, Mean (SD)	3.750 (0.521)	3.570 (0.480)	**<0.0001**
Hours Attempted Diff., Mean (SD)	0.146 (2.210)	-0.125 (2.310)	**0.0400**
Gender: Male, %	43.09%	54.79%	**<0.0001**
Rural: Yes, %	20.72%	25.17%	0.0700
Residency: Out-Of-State, %	39.19%	28.60%	**0.0001**
FA19 Enroll. Status:			
Continuing, %	44.44%	50.86%	**0.0300**
New Freshman, %	50.60%	46.40%	0.2000
New Transfer, %	4.96%	2.74%	0.0600
Online Experience: Yes, %	23.87%	25.51%	0.5000

*P-value from a two-sample t-test for comparing means or a *χ*^2^ test for comparing proportions.

The aggregate regression results obtained from 100 completed (imputed) datasets (see the Statistical Analysis section for details of imputation) are reported in Table 6. Comparing these results to the respondents only model results (see Full Sample Model I in Table 2), we notice general agreement between the two sets of analyses. Like the respondents only model, students’ university experience (FA19 Enroll Status) and perception about performance change due to the transition to ERI (Performance Percept.) are significant predictors of GPA Change (SP20 –FA19) in the completed-data model. Specifically, both models show that compared to continued students, students classified as new freshmen or new transfer in Fall 2019 experienced higher drops, on average, in their GPA after the transition to ERI. Also, both models show that students who perceived decline in their performance after transition to ERI had higher drops, on average, in their GPA than other students. One discrepancy between the results of the two models is that standardized ACT/SAT score was significant in the respondents only model (p-value = 0.0038) but not in the completed-data model (p-value = 0.6292), whereas weighted high-school GPA (HS GPA) was insignificant in the respondents only model (p-value = 0.5758) but became significant in the completed-data model (p-value = 0.0030). Both variables were included in the models to account for pre-college preparation level of students. Finally, while residency (in-state vs out-of-state) was not statistically significant in the respondents only model it became marginally significant (p-value = 0.0451) in the completed-data model.

**Table 6:** Regression analysis results aggregated over *m =* 100 completed (imputed) datasets.

Explanatory Variable	*n =* 1250			
	Coefficient	Lower 95% CI	Upper 95% CI	P-value
ACT/SAT	0.014	-0.042	0.070	0.6292
HS GPA	0.158	0.054	0.262	**0.0030**
Gender: Male	-0.095	-0.193	0.003	0.0583
Rural: Yes	-0.046	-0.167	0.075	0.4583
Residency: Out-Of-State	0.107	0.002	0.211	**0.0451**
Living On-campus: Yes	0.059	-0.106	0.225	0.4812
Living Place Safety	0.068	-0.040	0.177	0.2172
Financial Aid SP20: Yes	0.182	-0.015	0.379	0.0699
FA19 Enroll. Status:				
Continuing (Ref.)				
New Freshman	-0.234	-0.343	-0.124	**<0.0001**
New Transfer	-0.345	-0.582	-0.107	**0.0045**
Online Experience: Yes	-0.044	-0.167	0.079	0.4850
Instructors Support	0.031	-0.037	0.100	0.3685
Study Environment:				
Didn’t change (Ref.)				
Improved	-0.144	-0.396	0.108	0.2628
Worsened	0.045	-0.107	0.196	0.5623
Financial Situation:				
Didn’t change (Ref.)				
Improved	-0.239	-0.481	0.003	0.0531
Worsened	-0.001	-0.124	0.121	0.9809
Performance Percept.:				
Didn’t change (Ref.)				
Improved	0.031	-0.164	0.226	0.7521
Worsened	-0.254	-0.402	-0.106	**0.0008**
Hours Attempted Diff.	-0.003	-0.023	0.018	0.8092
Internet Access: Yes	-0.071	-0.296	0.154	0.5359
Tech Availability:				
Had All Tech (Ref.)				
Purchased Tech	0.030	-0.146	0.206	0.7407
Couldn’t Acquire Tech	0.104	-0.077	0.286	0.2574
Infected By COVID:				
None (Ref.)				
Close Friend(s)	-0.070	-0.281	0.140	0.5111
Family Member(s)	0.034	-0.139	0.206	0.7023
Both	-0.061	-0.298	0.176	0.6140
Prefer Not to Answer	0.053	-0.219	0.324	0.7025
Died By COVID:				
None (Ref.)				
Close Friend(s)	-0.261	-0.626	0.105	0.1621
Family Member(s)	0.011	-0.232	0.253	0.9318
Both	0.057	-0.486	0.600	0.8353
Prefer Not to Answer	-0.195	-0.480	0.090	0.1802
COVID News:				
<0.5 hour (Ref.)				
0.5–1 hour	-0.123	-0.279	0.032	0.1188
1–2 hours	0.028	-0.140	0.197	0.7403
2–3 hours	0.002	-0.204	0.209	0.9839
>3 hours	0.071	-0.117	0.260	0.4575
Constant	-0.980	-1.700	-0.259	**0.0078**

## Discussion

Several of the issues that students encountered during the transition to emergency remote instruction (ERI) due to the COVID-19 outbreak were identified and discussed. In this study, the authors investigated the impacts of the transition to emergency remote instruction (ERI) due to the COVID-19 outbreak on student performance. While accounting for many covariates, the study focused on examining three specific factors that were hypothesized to explain/correlate with these variations: namely, students’ enrollment status (first-year, new transfer, and continuing), students’ previous online course experience, and students’ personal experience with the COVID-19 (i.e., having family members or close friends infected by or dying from COVID-19). One of the results indicated that continuing students were able to maintain significantly better overall performance (as measured by change in GPA between Spring 2020 and Fall 2019) than first-year (freshman/transfer) students. The finding of the continuing students significantly outperforming the first-year students (freshman/transfer) suggests that our institutions of higher education need to pay special attention to these first-year students, especially at the times of shifts in instruction delivery methods. In addition, this finding goes along with the result reported by Cejda et al. [27] who reported on “transfer shock” which can vary across disciplines with a decrease in grade performance for STEM disciplines. It appears that the transfer students at the current university experienced “transfer shock” to a certain degree, and university personnel need to work to acclimate these students to the university as soon as possible.

Another result of the current study is that those students with previous online course experience were not significantly different from those without such experience. Even though the current study found that those without prior online learning experience did not seem to be adversely impacted, this finding contradicted that finding of Hachey et al. [39] who found that students with successful previous online experience have better success in subsequent online courses. Therefore, providing the students with meaningful online learning experiences may improve their online learning experiences later. In addition, the literature suggests that prior experience tends to improve one’s self-efficacy and improving self-efficacy improves performance.

The third result is that those students who had close friends or family members that contracted COVID-19 were not significantly different from those without such experiences. This finding differs from that found by Servaty-Seib and Hamilton [45] who found that students’ GPA tends to be negatively affected during the semester of their loss.

**Fig 5:**
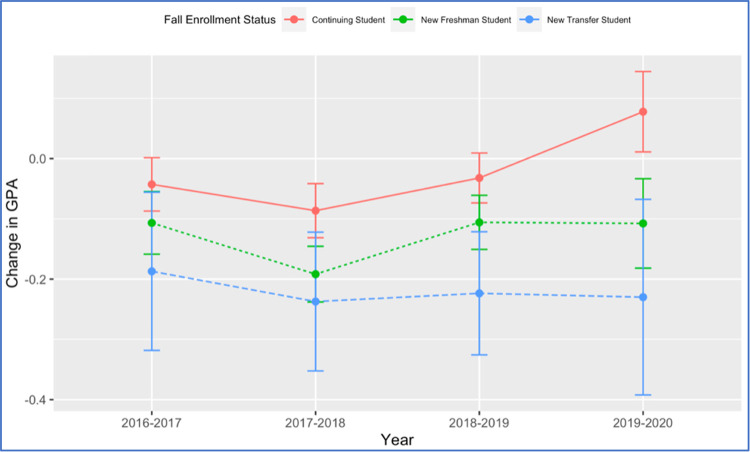
Comparing GPA change (SP—FA) by fall enrollment status over four academic years. The line segments represent 95% confidence intervals around the means represented by closed circles.

Students’ data from the years prior to the pandemic year (2016–2019) show that the performance gap between first year and continuing students, although has been emphasized by the pandemic, is not unique to the pandemic year. It is readily seen from Fig 5 that prior to the pandemic year, first-year students were usually more likely to experience higher drops in their performance between Fall and Spring semesters than continuing students, with all students experiencing some drop in their performance (95% confidence intervals below zero). While first-year students continued to experience drops in performance from Fall 2019 to Spring 2020 similar to those drops seen between Fall 2018 to Spring 2019, continuing students were able to improve their performance between Fall 2019 to Spring 2020 as shown by the entirely positive 95% confidence interval. These results indicate that both first year and continuing students were able to adjust for the learning challenges induced by the COVID-19 pandemic, with continuing students even being able to utilize the changes imposed by COVID-19 to improve their performance. These results are consistent, at least to some extent, with other results in the literature that found a significant positive correlation between the COVID-19 confinement and students’ performance (e.g., [3]).

### Limitations

This is a cross-sectional study that provides a snapshot of the impact of COVID-19 induced campus closure and emergency remote instruction (ERI) on the performance of underrepresented minority students. Thus, this study cannot establish causality, but it helps to understand the variations in students’ experiences and performance during the transition to ERI and to identify the factors that explain with these variations. The study did not employ a carefully designed probability sample for data collection. Instead, a convenience sample of students enrolled in lower- and mid-level mathematics/statistics courses was used to quickly collect the study data prior to the spring 2020 semester ended. Since the sample size was sufficiently large, this non-probability sample is sufficient for this kind of exploratory study, but caution should be taken when considering the generalizability of the study results. Finally, the study could have benefited from data on students’ course engagement and activity before and after the transition to ERI to explore the impact of the transition on students’ involvement in the course. However, due to many of the courses being taught face-to-face prior to the pandemic, this data was not available for many of the courses from which students were recruited to take part in the study.

### Implications

Studying the influence of the COVID-19 pandemic on higher education has received considerable attention from many researchers soon after the start of the pandemic leading to several research studies examining the impact of the pandemic-induced school closure and ERI on the various aspects of students’ academic life. The current study added to this body of literature by documenting the varying experiences of underrepresented minority students during the transition to ERI and by examining the association between ERI and students’ performance. The current study extended the main finding in the literature, which is a significant positive correlation between the COVID-19 confinement and students’ performance, to underrepresented minority students. Additionally, through investigating the differences in first year and continuing students’ performance between the fall semester and spring semester, the study suggests that extra efforts are needed to support first-year students and transfer students to successfully transition from their first semester at the university to their second semester while maintaining the same GPA or improving it.

The current study motivates several related future studies. A study expanding the current work to include more data from other semesters under the COVID-19 restrictions (e.g., Fall 2020 and Spring 2021) shall improve our understanding of the influence of the pandemic on the performance of underrepresented minority students. To understand the long-term impact of ERI on students’ performance, another study should investigate the students’ performance in subsequent courses (e.g., performance in the second Calculus course after the first Calculus course) after their transition to ERI in Spring 2020.

## Conclusion

The present study analyzed the effect of emergency remote teaching on students’ performance during the coronavirus pandemic. The study of the effects of the COVID-19 pandemic has served as the focus of many educational research studies. Our research study, despite the identified limitations such as being limited to one university, has the potential to broaden the knowledge base on this topic. When considering expanding this study to other universities, the results may be different. The setting for this study is a university whose population is predominantly black American and originates from a state located in the Southeastern United States. Many of the students at the current university found themselves dealing with distractions to their online learning such as working full-time positions to support their families. Conducting this study at another university depending on the location may bring students with educational backgrounds and experiences much different from those students at the present university who may not have to endure as many distractions. It would be interesting to investigate these research questions at another university to determine if the findings would be the same.

Our study found that the continuing students outperformed first-year students, including first-year students and transfer students between Spring 2020 and Fall 2019, as measured by GPA. The pandemic has emphasized this gap, but after analyzing data from prior years, it is not unique to the pandemic. This finding lends itself to further research in terms of understanding why this drop in GPA is occurring and what can be done to prevent it. Proceeding with this knowledge, university administrators and faculty can develop an intervention focused on preventing that drop in GPA from occurring for first-year students. This intervention could help in improving the university retention rate and serve to encourage our first-year students. Most faculty and university administrators want students to be encouraged and have a positive experience.

This study also found that students who did not have prior online learning experience were statistically the same as those students who did have prior online learning experience and that the performance of students who had close friends or family who were sick or died from the coronavirus was not significantly different from those who did not close friends or family affected by the coronavirus. These findings are worthy of additional study because we want to confirm that this result will be the same across other universities. In addition, these findings could also benefit from pursuing future studies at our own university with additional data from future semesters. As we continue our efforts, we plan to continue with a research agenda that can further confirm these results.
